# An Unusual Back Muscle Identified Bilaterally: Case Report

**DOI:** 10.7759/cureus.2816

**Published:** 2018-06-15

**Authors:** Juan J Altafulla, Mayank Patel, R. Shane Tubbs, Joe Iwanaga, Zachary Litvack

**Affiliations:** 1 SNI, Seattle Science Foundation, Seattle, USA; 2 Clinical Anatomy Research, Seattle Science Foundation, Seattle, USA; 3 Neurosurgery, Seattle Science Foundation, Seattle, USA; 4 Seattle Science Foundation, Seattle, USA; 5 Neurosurgery, Swedish Neuroscience Institute, Seattle, USA

**Keywords:** variation, ribs, respiration, anomaly, myology, serratus posterior superior

## Abstract

Most muscular structures in the human body are named based on their function, origin/insertion, or shape. During routine dissection of the back, an unusual muscle was found deep to the rhomboid muscles. The details of this case and a review of the extant literature are provided.

## Introduction

The serratus posterior superior (SPS) muscle arises as a thin aponeurosis from the lower part of the nuchal ligament, the spinous processes of the seventh cervical, and first two thoracic vertebrae and adjacent supraspinous ligaments [1]. It follows a lateral course ending as four digitations that insert lateral to the angle of the third, fourth, and fifth ribs [2]. Traditionally, the SPS and the serratus posterior inferior (SPI) have been thought to act as accessory respiratory muscles [3, 4]. However, this function has not been supported by electromyography studies [5, 6].

Herein, we report the bilateral occurrence of an unusual muscle of the back found during routine dissection and compare it to other reports in the literature.

## Case presentation

**Figure 1:**
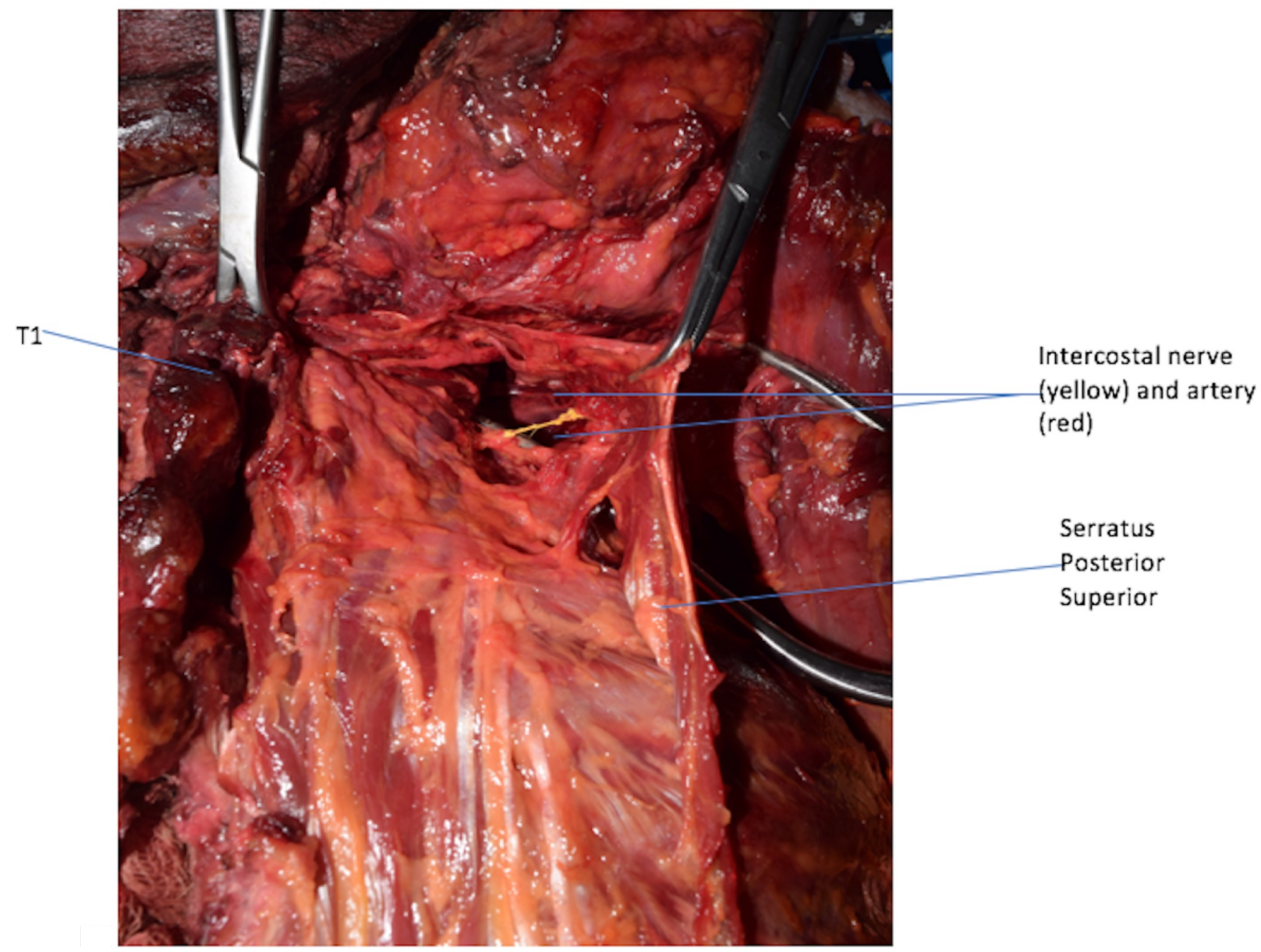
Serratus Posterior Superior. Serratus Posterior Superior with its underlying vascular and nerve supply. Left: medial; right: lateral; up: cephalic; down: caudal.

During routine dissection of a male cadaver aged 59-year-old at death, an unusual muscle was identified on the back. The muscle was deep to the rhomboids, superficial to the erector spinae and was more or less vertically arranged. The origin of the muscle was from the spinous processes of the lower cervical vertebrae and the insertion was onto the second through sixth ribs (Figure 1). The innervation and blood supply were via the intercostal nerve and artery, respectively.

**Figure 2:**
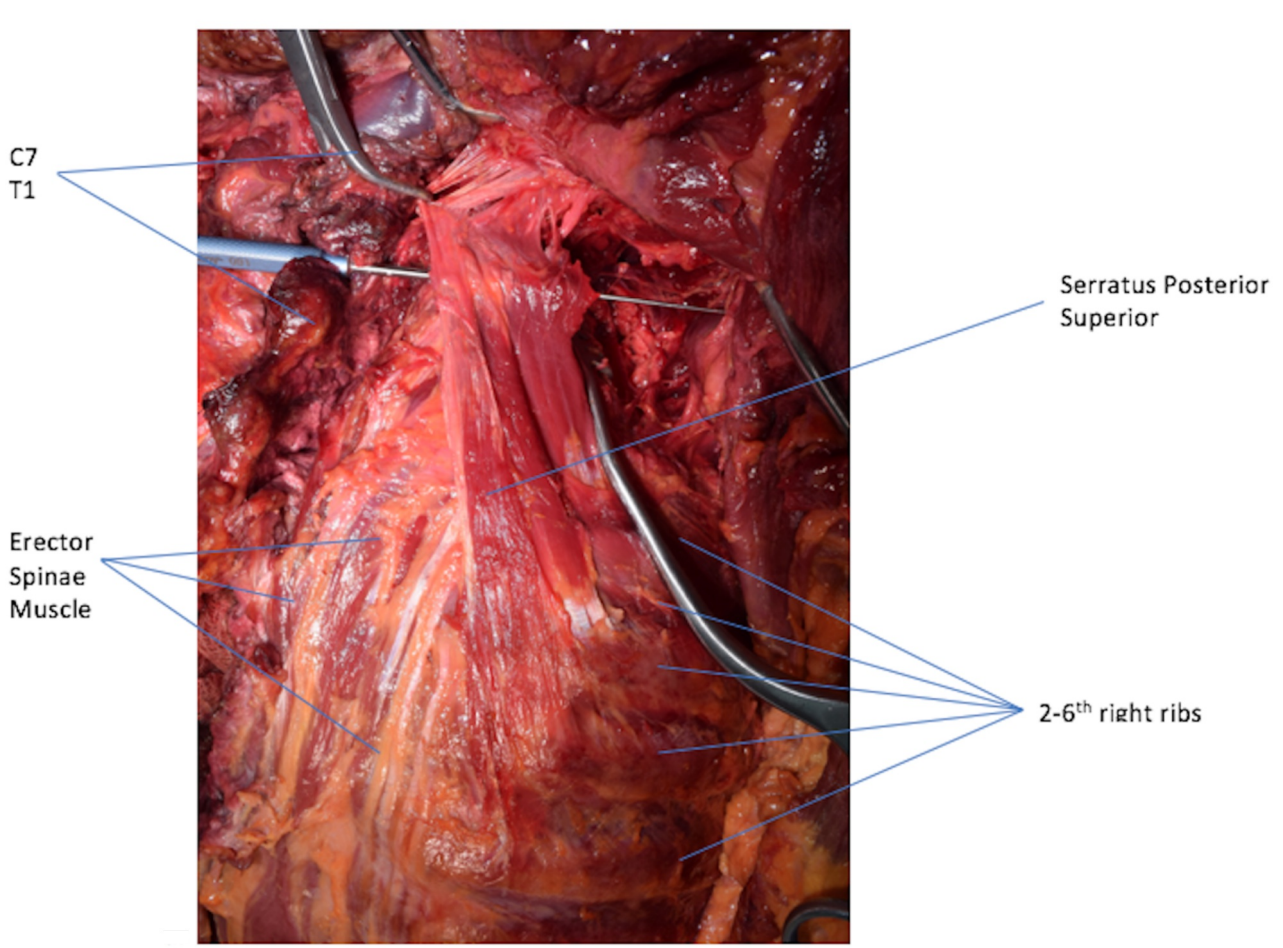
Back Muscles. Serratus Posterior Superior running in a vertical fashion. Left: medial; right: lateral; up: cephalic; down: caudal.

Although the fiber direction and number of rib attachments were not consistent with the SPS, the position of the muscle between the rhomboids and erector spinae indicated that this muscle most likely represented an unusual variation of the SPS (Figure 2). No other anatomical variations were found on the back and no pathology such as scoliosis was identified.

## Discussion

The present case depicts a variation of one of the back muscles, the SPS. A small number of variations have been reported in the literature for the serratus posterior muscles with most of these in regard to the number of ribs that they attach to. Most cases have reported a range from three to six digitations that can extend from as high as C4 all the way down to T5 [1]. Rarely, the SPS can be absent or have attachment to other regional muscles such as the erector spinae [1]. Typically, this muscle has an oblique orientation. However, in our case, the fibers of the muscle were more or less vertical in nature.

Multiple functions have been attributed to the SPS and SPI from aiding in inspiration to having a proprioceptive function [7]. Regarding a respiratory function, Loukas et al. [8] evaluated the SPS and SPI in 50 adult cadavers. Eighteen of the specimens had a history of chronic obstructive pulmonary disease. However, in this latter cohort, there were no statistically significant differences between sides, race, sex, or age in regard to the dimensions of these muscles. As one would expect hypertrophy of accessory respiratory muscles in patients with obstructive respiratory disease, these authors concluded that based on their anatomical study, the SPS and SPI are not involved in respiration.

Additionally, improved imaging modalities and embryological knowledge will hopefully, in the future, better elucidate the function of such muscle variations of the back [9-13].

## Conclusions

Although it is not clear what function such a thin, vertically arranged muscle might have, as seen in our case, documenting such muscular variations is important from an archival and future study perspective.
